# Deficiencies in culturally competent asthma care for ethnic minority children: a qualitative assessment among care providers

**DOI:** 10.1186/1471-2431-12-47

**Published:** 2012-07-10

**Authors:** Conny Seeleman, Karien Stronks, Wim van Aalderen, Marie-Louise Essink Bot

**Affiliations:** 1 Department of Public Health, Academic Medical Centre/University of Amsterdam, PO Box 22660, 1100, DD, Amsterdam, the Netherlands; 2 Department of Paediatric Pulmonology, Emma Children's Hospital, Academic Medical Centre/University of Amsterdam, PO Box 22660, 1100, DD, Amsterdam, the Netherlands

## Abstract

**Background:**

Asthma outcomes are generally worse for ethnic minority children. Cultural competence training is an instrument for improving healthcare for ethnic minority patients. To develop effective training, we explored the mechanisms in paediatric asthma care for ethnic minority patients that lead to deficiencies in the care process.

**Methods:**

We conducted semi-structured interviews on care for ethnic minority children with asthma (aged 4-10 years) with paediatricians (n = 13) and nurses (n = 3) in three hospitals. Interviews were analysed qualitatively with a framework method, using a cultural competence model.

**Results:**

Respondents mentioned patient non-adherence as the central problem in asthma care. They related non-adherence in children from ethnic minority backgrounds to social context factors, difficulties in understanding the chronic nature of asthma, and parents’ language barriers. Reactions reported by respondents to patients’ non-adherence included retrieving additional information, providing biomedical information, occasionally providing referrals for social context issues, and using informal interpreters.

**Conclusions:**

This study provides keys to improve the quality of specialist paediatric asthma care to ethnic minority children, mainly related to non-adherence. Care providers do not consciously recognise all the mechanisms that lead to deficiencies in culturally competent asthma care they provide to ethnic minority children (e.g. communicating mainly from a biomedical perspective and using mostly informal interpreters). Therefore, the learning objectives of cultural competence training should reflect issues that care providers are aware of as well as issues they are unaware of.

## Background

Asthma outcomes are generally worse for children from ethnic minority backgrounds [1-3]. A recent study in the Netherlands showed that ethnic minority children have poorer asthma control and more complaints than their Dutch peers [4]. This reflects a significant, avoidable burden of illness, because asthma prevalence is as high as 5% to 10% among children in Western societies [5], and ethnic diversity has increased among young Western populations [6,7].

Low adherence to preventive treatment with daily inhalation corticosteroids (ICSs) is an important barrier to achieving optimal asthma control [8,9]. Paediatric asthma care focuses on parents’ self-management of their children’s asthma. Rather than parents having full responsibility, an effective partnership between the care provider and the child’s parents is considered a key component for effective self-management [10]. Therefore, care providers are also responsible for this. However, research has found more misunderstandings and less patient satisfaction in medical consultations with ethnic minority patients. Problems arise from cultural differences, linguistic barriers, and perceptual bias [11].

Cultural competence is regarded as an instrument for improving quality of care for ethnic minority patients, and is generally defined as the combination of knowledge, attitudes, and skills necessary for delivering high-quality care to an ethnically diverse patient population [12,13]. Training can make care providers aware of ethnic differences and improve their cultural competence. However, to develop effective, informative training, we first have to gain insight into the daily practice of specialist paediatric asthma care for ethnic minority patients. Based on such insights, learning objectives can be defined for cultural competence training targeted specifically at such care.

The aim of this qualitative study was to explore those mechanisms in paediatric asthma care that lead to deficiencies in the care process for ethnic minority patients. The results will be used to develop cultural competence training for specialist paediatric asthma care providers.

## Methods

### Design

We conducted semi-structured qualitative interviews with care providers in specialist paediatric asthma care that focussed on their own experiences and reported concrete behaviour with children from ethnic minority groups with asthma (aged 4–10 years) and their parents. We defined “children from ethnic minority groups” as children who had at least one parent who was born abroad [14].

We chose to conduct individual, qualitative interviews because of its appropriateness to provide an in-depth understanding of the care process for ethnic minority children with asthma. Although Dutch law (the Medical Research Involving Human Subjects Act) did not require ethical approval for this interview study, each respondent was adequately informed of the aims and methods of the study and we took every precaution to guarantee the respondents’ anonymity.

### Respondents

We established a purposive sample of nine paediatricians (two male, seven female) and four paediatric pulmonologists (three male, one female) in three hospitals in Amsterdam (two paediatric university hospitals, one general inner-city teaching hospital). Their experience in working as paediatricians varied between <10 years (n = 7), 10-20 years (n = 3) and >20 years (n = 3). We chose Amsterdam because over 50% of its young population (aged 0-15) is from non-Western ethnic minority backgrounds [7]. Although over 170 nationalities live in Amsterdam, the largest ethnic groups are Moroccans and Turks (both ethnic groups have a history in labour migration), and Surinamese (Suriname is a former Dutch colony in South America) [15].

Eight physicians worked in an academic setting, and five in the general hospital. We chose these different settings to ensure variation in ethnic patient population, provider population, asthma severity, and type of healthcare organisation. Additionally, an asthma nurse was interviewed in each hospital (all female). Respondents were selected because they worked at one of the three hospitals’ paediatrics department at the time of the study. They were approached by the first author (CS). Three care providers declined an interview: two because of time constraints and one because of illness. We stopped approaching care providers when interviews revealed no new information (saturation of data).

### Data collection

We used a semi-structured topic list based on issues of specific importance in asthma care in an ethnically diverse healthcare setting (Table 1). We asked respondents to elaborate on a specific case from their own daily practice. Interviews were conducted by CS who is an independent researcher and not involved in patient care, and took between 40 and 75 minutes*.* All interviews were audiotaped and transcribed verbatim.

**Table 1:** Topic list

**Topic**	**Interview**
Provider’s experience	Experience in caring for children from ethnic minorities in general
A specific case	Elaboration of a specific case (“Please think of a case of a patient with asthma from an ethnic minority background (aged 4-10) that did not go smoothly in your opinion.”)
	‒ Case background
	‒ Medication
	‒ Advice other than on medication
	‒ Self-management
	‒ Consultation
	*(The interview focused on “What happened?” and “What did you do/how did you react?”)*
Themes related to culturally competent care	- Working with interpreters
	- Eliciting patients’ perception on asthma/medication
	- Influence of social context
Improving care	Respondents’ ideas about specific requirements for improving care for children from ethnic minority backgrounds with asthma

### Data analysis

We used a framework approach to analyse the transcripts [16]. After familiarising ourselves with the data, we (CS and MLE-B) identified a coding framework based on Seeleman’s cultural competence model [13]. Seeleman’s model identified the important aspects in providing care to ethnic minority patients, including the influence of culture on the care process, patients’ social context, stereotyping and prejudice, and communication barriers. These issues were all identified within the coding framework. Transcripts were coded by CS according to this framework. Subsequently, the data was charted according to two parallel charts: 1) What are the care provider’s experiences? and 2) What actions does he or she take in such situations? These charts were used to explore mechanisms in the care process that may lead to deficiencies in culturally competent asthma care for ethnic minority children. For further refinement, CS and MLE-B discussed this analysis extensively until consensus on interpretation was reached.

## Results

### Central problem: non-adherence

Non-adherence (especially to daily preventive ICSs but also to lifestyle recommendations) is generally known to be the major problem in paediatric asthma care, and respondents in our study identified issues that add on non-adherence in asthma care to ethnic minority children.

The first section describes three factors of influence on adherence in patients from ethnic minority backgrounds as mentioned by the respondents: social context, illness perception, and language. The second section describes the respondents’ reported reactions to patients’ non-adherence. Quotes are presented throughout the results to illustrate the findings.

### Factors of influence on adherence in ethnic minority patients

#### Social context

Respondents related children’s adherence to families’ social context. Several respondents described multi-problem families in which various problems coexisted: a sick parent, problems in the parents’ relationship, and parenting problems. These problems affected children’s adherence in different ways. Sometimes the responsible parent was so caught up in his or her own problems that there was barely any attention left for the child’s asthma. Or a parent had difficulties getting a child to take the medication, while other parents shifted too much responsibility onto the child. The following case, described by one of the respondents, illustrates these issues:

"“Well, the family has three children, of which two suffer from a severe allergy and asthma, exercise-induced asthma and allergic asthma. <… > divorced parents of, well they actually are very lively boys. The mother gives an impression of being overburdened. Youth care and pedagogic support has been offered in the past and it seems to be going better now. And then you have the oldest, he is kind of entering puberty and he believes it is nonsense that he has to use inhalation steroids. He just is not motivated himself so the mother tries to do encourage him, but it just does not work.” (A6)"

#### Illness perception: chronicity of asthma

Respondents also ascribed non-adherence to different illness perspectives. Some respondents merely said that parents from ethnic minority backgrounds “perceive their child’s illness differently”; others had more concrete ideas of differences in illness perception. They expressed more difficulties explaining the chronic nature of asthma to parents from ethnic minority backgrounds, and found it harder to get them to understand the consequences this has for daily life. The following respondent related these differences to culture:

"“I think that might be the biggest problem, letting parents and patients know the disease can last a lifetime and cause lots of symptoms, and that the doctor can’t cure the disease, but can relieve symptoms. I think that might be the cultural difference, I don’t know exactly.” (A9)"

This was also explained as a “phase” difference – a phase Dutch society (and patients) have already gone through, and patients from minority backgrounds have not:

"“Here we’ve already gone through all of that, we’re talking about 25, 30 years back, gone from, say, the acute illness concept and getting better to a concept of, it’s a chronic disorder, you have to take care of it in the meantime.” (A4)"

Furthermore, respondents indicated that differences in perceiving the chronic nature of asthma create the unmet expectation that asthma can be cured:

"“Well, there is a cultural difference, insofar as particularly in the Moroccan community, quick diagnosis, treatment, [is expected] and everything has to be alright again, they want to see a plan, a course of action.” (A12)"

#### Language

Although respondents did not label language barriers as a large problem, at the same time “language difference” was described as a factor that hindered parents’ understanding of asthma and its treatment. Respondents explained that the message they generally communicate (which they described as “what asthma is and what do you have to do about it”) was a complex one.

Care providers also mentioned that a language barrier made it more difficult for them to assess whether their information was well understood:

"“And I think if you speak in your own language it’s much easier to know whether something has really gotten through or if we just have to do it again, or do it differently.” (A4)"

### Reported reactions to patients’ presumed non-adherence

In this section we describe the reactions reported by respondents to patients’ non-adherence, including retrieving information, providing information, occasionally providing referrals for social context issues, and using informal interpreters.

#### Retrieving additional specific information

Care providers need to get a clear picture of what is going wrong, and therefore expressed a need for specific information from patient and parents on the child’s health situation and the extent of and reasons for non-adherence. Respondents experienced more barriers in gathering such information from ethnic minority patients due to two mechanisms: communication barriers and a discrepancy in care providers’ information needs and patients’ daily lives.

#### Communication barriers

Respondents described various strategies for retrieving information, including additional questioning, asking questions in an empathic way (“It’s very difficult to get a child to take his medication every day – how is that for you?”), or asking children directly instead of parents. These techniques did not always yield the desired information. Respondents recognised specific communication patterns with ethnic minority parents, including “yea-saying”, i.e. socially desirable response behaviour. In their eyes, these patterns hindered information retrieval. Keeping an asthma diary as an alternative source of information also caused difficulties because of functional illiteracy and language barriers.

#### Discrepancy in care providers’ information needs and patients’ daily lives

Sometimes the type of information care providers required did not seem to be in keeping with patients’ daily lives. Respondents expressed the feeling that their questions did not yield the “right” information. A respondent explained that the information physicians are looking for is very concrete: how often and how much medication was used during which time span? Whereas Dutch parents are usually able to answer these questions, with ethnic minority patients this kind of information seems harder to retrieve. This respondent believed that giving medication in ethnic minority families is something that blends into the day rather than something that happens according to a schedule and that can easily be reproduced when a care provider asks for it.

Some respondents explained that in cases where they lacked insight into home situations, they tried to monitor the patient more closely (e.g. by being more prescriptive in their treatment recommendations instead of stimulating self-management).

#### Providing biomedical information

Providing information was our respondents’ strategy of choice for improving adherence. They focused on transferring biomedical knowledge (sometimes repeatedly) to help parents understand their child’s medical situation.

"“Yes, I explain it again, sometimes I look like a fool but yes, I make another drawing, your windpipe is constricted, how can you prevent this. And I explain the risks again, particularly to the mother. That we see children in the ICU, on the respirator, just because they’re not compliant.” (A6)"

Some explained that they tried to connect with what parents put forward during the consultation:

"“If someone consistently finds these attacks distressing, that you follow this with your information, like, yes, but this is especially to prevent these attacks.” (A12)"

The respondents explained they use drawings or anatomical models to visualise the information for parents. Or they compared the “invisible” (inflammation of lung tissue) with something “visible” (like eczema on the skin). Almost all paediatricians mentioned a preference for referring patients to an asthma nurse for more detailed asthma education. Which patients were actually referred differed per healthcare organisation (e.g. all new patients, patients with uncontrolled asthma, or patients who had been prescribed new medications).

One care provider used a specific metaphor to get information across:

##### The story of the cowboy

A cowboy has two things. He has a horse, which is reddish brown (a colour similar to the corticosteroid inhaler) and he has a pistol, which is steel blue (a colour similar to the Ventolin inhaler). A good cowboy looks after his horse twice a day and only shoots when he has to. If the cowboy only wants to take care of his horse when he really needs one, you can imagine the horse will be dead by then. That doesn’t work. And that doesn’t work for the medicine either. (A10)

#### Social context issues: occasional referrals

In cases where social context issues such as a parent’s illness or parenting problems influenced a child’s adherence, respondents explained they find it important to acknowledge the problem to parents, but they doubted the extent to which they could support them in these issues. Although in some cases they referred parents for psychosocial support, these referrals were not formalised in protocols.

#### Using informal interpreters

Often, respondents associated patients from ethnic minority backgrounds with language barriers. Although respondents regarded language barriers as posing a risk to transferring and gathering information and therefore posing a risk to adherence, the general opinion was that “the language problem wasn’t so bad.” In daily practice, respondents seldom used formal interpreters (which was free of charge in the Netherlands). Respondents mentioned several barriers that hampered communication when using formal interpreters: it takes too much time to request an interpreter, the conversation gets impersonal with an interpreter on the phone, and the conversation is reduced to one-liners. The respondents regularly used informal interpreters (like family and friends) brought in by parents. These conversations proceeded naturally, and informal interpreters were given no instructions on how the respondents would like them to translate the conversation. Respondents were aware of risks that may arise from using informal interpreters, but saw little danger in using them in standard asthma care:

"“Usually it < the patient bringing an informal interpreter > is just convenient. It depends on the type of conversation, but the type of conversation we have here in the outpatients’ clinic, that is rarely so emotionally charged that it would be a problem.”(A12)"

## Discussion

In this study we showed that mechanisms in paediatric asthma care that lead to deficiencies in the care process for ethnic minority children were mainly related to non-adherence. It seems that most factors our respondents discussed (such as influence of social context factors and difficulties in understanding asthma as a chronic disease) are not related to ethnic minority patients in particular. Rather, they emphasise certain aspects of asthma care that are likely to create problems in the general patient population.

Educational literature describes different stages of competence. In the stage “conscious incompetence,” care providers recognise mistakes and difficulties in their actions, and will be able to report them. However, this stage is preceded by the stage of “unconscious incompetence”: a stage in which learners (or care providers) are unaware of their incompetence [17] and therefore cannot report them. If applied to our study, this theory means that apart from the difficulties care providers reported (and were aware of), difficulties they were unaware of might also complicate the care process.

A first difficulty the respondents seemed unaware of is related to providing information. Providing information was our respondents’ strategy of choice in case of non-adherence, with the intention of increasing parents’ knowledge on asthma. Studies have shown that medical information is generally not easily understood by patients due to such things as unfamiliarity with medical technical terminology [18,19]. Low health literacy (the degree to which individuals have the capacity to obtain, process, and understand basic health information [20]) complicates patients’ understanding: low parental literacy has been associated with poorer asthma outcomes [21]. For effective communication, it is recommended that care providers make their own language accessible by avoiding technical jargon and using plain language instead [22]. Low health literacy is more prevalent in minority populations [23]; however, our respondents did not reflect on their own use of technical jargon and how that might impede communication.

A second difficulty respondents seemed unaware of is related to language. Respondents recognised language difficulties as a barrier to information transference. Respondents explained that language barriers were overcome by using informal interpreters instead of formal ones. However, research has shown that using professional interpreters in healthcare has added value over the use of informal interpreters [24] and is therefore preferred if there are language barriers.

Respondents indicated that different illness perspectives were related to non-adherence. Kaptein et al. [25] showed the importance of patients’ perceptions of disease and treatment for asthma outcomes. The idea of “no symptoms, no asthma” (when patients consider asthma to be an acute rather than a chronic illness) is found repeatedly, irrespective of ethnicity [25,26]. Parents who do not perceive asthma as a chronic disease are more likely to administer medication only when the child experiences symptoms. Our respondents did not seem to regard discussing illness perceptions as a standard part of consultations. In cultural competence literature, though, exploring patients’ illness perspectives is considered a central aspect of culturally competent care [27], because culture has a strong influence on illness perspectives [28]. Rather than a biomedical communication style, a patient-centred one helps to get information about cultural differences, expectations, and influence of social context factors out in the open [27].

Self-reflection receives much attention in the literature on multicultural care [29]. This acknowledges the importance of reflecting on one’s own cultural background and assumptions, biases, and values, especially when taking care of people from other ethnic or cultural backgrounds [29,30]. The respondents reflected little on their own cultural and professional backgrounds. However, care providers did seem aware of the existence of stereotyping. During the interviews, statements like “The same goes for Dutch patients” or “This does not apply to all patients from that ethnic background” were made regularly.

To develop effective, meaningful cultural competence training for specialist paediatric asthma care providers, we have to turn our findings into learning objectives that reflect both the issues care providers were *aware* of as well as the issues they were *unaware* of. For care providers to adequately identify reasons for non-adherence in children with asthma from ethnic minority backgrounds, and to effectively act on these, we identified the following objectives:

Ability to use patient-centred communication skills in giving and retrieving information;

Awareness of different illness perceptions and ability to communicate effectively about this;

Ability to effectively overcome language and health literacy barriers;

Ability to reflect on one’s own background and stereotyping in intercultural contexts (Table 2).

**Table 2:** Difficulties in paediatric asthma care for ethnic minority children

Providers were aware of	a) complex social contexts
	b) difficulties in explaining the chronic nature of asthma and its consequences
	c) gathering information about the child
Providers were unaware of	d) communicating from a biomedical perspective (mainly providing knowledge)
	e) not giving attention to illness perceptions
	f) not using formal interpreters
	g) not adapting their own language (plain language instead of medical jargon)
	h) the impact of providers’ own backgrounds on the consultation
Cultural competence	- Ability to use patient-centred communication skills in giving and retrieving information (issues a,b,c,d,e,g)
	- Awareness of different illness perceptions and ability to communicate effectively about this *(issue e)*
	- Ability to effectively overcome language and health literacy barriers *(issues f,g)*
	- Ability to reflect on one’s own background and stereotyping in intercultural contexts *(issue h)*

Evaluation of care provided to ethnically diverse patients often showed a “magnifying glass effect” [31]: difficulties in the care process are revealed that are not unique to patients from these groups but are more intense expressions of general paediatric care problems. For instance, if care providers communicate from a biomedical perspective, it is hard for all patients with low health literacy to understand the information they receive. Since health literacy skills in ethnic minority patients are generally lower, and language barriers might further complicate the communication, the negative effect on patients from ethnic minority backgrounds is larger. Because of the accumulation of characteristics that complicate care, ethnic minority patients experience more disadvantages from suboptimal care. The magnifying glass effect explains why the learning objectives we defined are not so “cultural” either. For the most part they are specifications of competences care providers should already possess. The most striking example is the importance of the ability to use patient-centred communication skills.

By using a qualitative research method, we obtained insight into issues respondents themselves related to adherence in children from ethnic minority backgrounds. By putting respondents’ experiences in the context of the general literature on asthma care and cultural competence, difficulties respondents were unaware of also became apparent.

Research on care providers’ cultural competence commonly uses instruments to measure their self-perceived cultural competence [32-34]. However, because care providers cannot report explicitly on things they are unaware of, some issues will go unnoticed. Although our interviews showed an extra dimension in the care process, other methods such as direct observation will provide added value in gaining full insight into the relationship between care providers and patients. Now we had to rely on care providers’ recall. Respondents were asked to elaborate on a specific case from their own practice in which they had experienced difficulties, to get insight in care providers’ concrete experiences. The examples discussed might not be representative for every day practice and therefore not reflect a general need for cultural competence training among these providers. Although during the interviews the issues discussed were placed in broader context (‘was this case exceptional or do these issues happen more often?’), methods like direct observation would provide a more detailed, objective insight into what actually happens during a consultation. Additionally, insight into patient experiences would provide information from their perspective.

A limitation of this study is the small number of interviewed respondents (n = 16). This was due to rapid saturation of the data. An explanation for limited variation in data and rapid saturation might be homogeneity of the respondent group. Although gender, years of experience and setting (academic/non-academic) did vary between respondents, we did not verify respondents’ country of birth. Based on their last names, mastery of Dutch language, and appearances, we assume they all had a western/an ethnic majority background. It might be that care providers from ethnic minority background would have put forward different experiences or communication styles during the interviews that would have diversified the data.

Developers of cultural competence training can use our findings as input for developing learning objectives. Although it is important to meet the educational needs of care providers when developing cultural competence training, we have shown it is equally important to take into account issues care providers are unaware of. However, care providers must first become aware of their “incompetence” before they will recognise their need to learn about these issues. We therefore recommend that creating awareness of providers’ “incompetence” should become a part of the training itself or a separate learning activity before the actual training.

Our study was limited to cultural competence at the level of care providers [35]. For providers to be able to provide culturally competent care, the healthcare organisation should provide the conditions necessary to enable care providers to work in a culturally competent way [36].

## Conclusions

This study provides keys to improve the quality of specialist paediatric asthma care to children from ethnic minority backgrounds. Importantly, we showed that care providers do not consciously recognise all the mechanisms that lead to deficiencies in culturally competent asthma care they provide to ethnic minority children (“unaware incompetence”). Therefore, the learning objectives of cultural competence training may need to start with issues that care providers are aware of, to get their interest. Once having them inside, cultural competence training should address unaware incompetence issues and change these into “awareness” and then into “competence” (e.g. the use of formal interpreters to overcome language barriers). Fortunately, our results also give reason to believe that if care providers continue to improve on the patient-centred skills they learn during medical training, they already “come a long way.”

## Competing interests

The authors declare that they have no competing interest.

## Authors’ contributions

KS, WvA and MLE-B wrote the grant application. KS, WvA, MLE-B and CS designed the study. CS collected the data. Data were analysed by CS, under supervision of MLE-B. CS drafted the article, all others contributed intellectual content to the paper, provided comments on subsequent drafts and approved of the final version. All authors read and approved the final manuscript.

## Pre-publication history

The pre-publication history for this paper can be accessed here:

http://www.biomedcentral.com/1471-2431/12/47/prepub
